# Role of caspases in CD95-induced biphasic activation of acid sphingomyelinase

**DOI:** 10.18632/oncotarget.15379

**Published:** 2017-02-16

**Authors:** Mario Stephan, Bärbel Edelmann, Supandi Winoto-Morbach, Ottmar Janssen, Uwe Bertsch, Cristiana Perrotta, Stefan Schütze, Jürgen Fritsch

**Affiliations:** ^1^ Institute of Immunology, Christian-Albrechts-University of Kiel, Kiel, Germany; ^2^ Department of Hematology and Oncology, University Hospital Magdeburg, Magdeburg, Germany; ^3^ Department of Biomedical and Clinical Sciences “Luigi Sacco” (DIBIC), Università degli Studi di Milano, Milano, Italy

**Keywords:** acid sphingomyelinase, ceramide, CD95 ligand, internalization, CD95-receptosomes

## Abstract

Acid sphingomyelinase (A-SMase) plays an important role in the initiation of CD95 signaling by forming ceramide-enriched membrane domains that enable clustering and activation of the death receptors. In TNF-R1 and TRAIL-R1/R2 signaling, A-SMase also contributes to the lysosomal apoptosis pathway triggered by receptor internalization. Here, we investigated the molecular mechanism of CD95-mediated A-SMase activation, demonstrating that A-SMase is located in internalized CD95-receptosomes and is activated by the CD95/CD95L complex in a biphasic manner.

Since several caspases have been described to be involved in the activation of A-SMase, we evaluated expression levels of caspase-8, caspase-7 and caspase-3 in CD95-receptosomes. The occurrence of cleaved caspase-8 correlated with the first peak of A-SMase activity and translocation of the A-SMase to the cell surface which could be blocked by the caspase-8 inhibitor IETD.

Inhibition of CD95-internalization selectively reduced the second phase of A-SMase activity, suggesting a fusion between internalized CD95-receptosomes and an intracellular vesicular pool of A-SMase. Further analysis demonstrated that caspase-7 activity correlates with the second phase of the A-SMase activity, whereas active caspase-3 is present at early and late internalization time points. Blocking caspases-7/ -3 by DEVD reduced the second phase of A-SMase activation in CD95-receptosomes suggesting the potential role of caspase-7 or -3 for late A-SMase activation.

In summary, we describe a biphasic A-SMase activation in CD95-receptosomes indicating (I.) a caspase-8 dependent translocation of A-SMase to plasma membrane and (II.) a caspase-7 and/or -3 dependent fusion of internalized CD95-receptosomes with intracellular A-SMase-containing vesicles.

## INTRODUCTION

CD95 is a member of the tumor necrosis factor receptor (TNF-R)-superfamily, and besides TNF-R1, one of the best characterized death receptors [1, 2]. CD95 exists as a membrane-bound form (mCD95) as well as a soluble version generated by alternative splicing or matrix metalloproteinase (MMP) mediated shedding. The soluble form of CD95 receptor is believed to have anti-apoptotic functions while the transmembrane receptor induces apoptosis, activation of and inflammation or acts as a costimulatory molecule [3–5]. Upregulation of CD95 in tumor cells is activated by various stimuli including TNF-α, IFN-γ, interleukins, nitric oxide, chemotherapy and ionizing radiation [6].

CD95 ligand (CD95L, FasL, CD128 or TNFSF6), is a type-2 transmembrane glycoprotein of the TNF-superfamily of cytokines, that activates apoptotic or non-apoptotic CD95 signaling pathways [7]. Membrane bound CD95L (mCD95L) of activated natural killer (NK) cells and T-lymphocytes induces apoptosis in infected or transformed cells by interacting with the mCD95 [8–10]. MMP 3, 7, 9 and ADAM-10 (a disintegrin and metallprotease-10) are able to shed m CD95L in different cellular systems releasing a soluble ligand (sCD95L) [11–14]. The soluble form competes with m CD95L for binding to the receptor and induces proliferation and cell migration but also inflammation rather than apoptosis [15–17]. For its physiological activity, in its extracellular part, CD95L contains a domain for self-aggregation and trimerization [18]. Apparently, coupling of two homotrimeric sCD95L molecules forms a hexameric structure which is able to induce apoptosis instead of proliferation [19].

Binding of CD95L to CD95-receptor can induce apoptotic and non-apoptotic signaling cascades. The decision, which pathway is activated, is not fully understood. The non-apoptotic pathway is preferentially activated when caspase-8 is inhibited through high levels of cFLIP (cellular FLICE/caspase 8-like inhibitory protein), followed by the recruitment of TRAF1 (TNF receptor associated factor 1), TRAF2, RIP (receptor-interacting protein) kinase and Raf-1 (rapidly accelerated fibrosarcoma) and resulting in ERK and NF-κB activation [20–22]. Apoptosis is induced by binding of the trimerized membrane CD95L resulting in receptor aggregation and a conformational change of its intracellular part [23]. Subsequently, the adaptor protein FADD (Fas associated protein with death domain) is recruited to the death domain (DD) of the receptor serving as a platform for the recruitment of caspase-8 to form the death inducing signaling complex (DISC). Active caspase-8 cleaves and activates effector caspases (e.g. caspase-3 or -7) leading to the induction of apoptosis [24].

In the context of CD95-mediated apoptosis induction, two differentially responding cell types have been described. In type I cells (e.g. SKW6.4, HuT78, ACHN), large DISCs are formed resulting in massive caspase-8 activation, and fast and direct induction of effector caspases and apoptosis. In type II cells (e.g. Jurkat, CEM, SR) only a small amount of DISCs are formed which results in less active caspase-8. To induce apoptosis, caspase-8 truncates Bid to form tBid which mediates the permeabilization of the mitochondrial membrane, the release of cytochrome c and the formation of an apoptosome [25, 26]. Active caspase-9 amplifies the apoptotic signal by activation of further effector caspases [27–29].

The internalization of CD95 is critical for apoptosis induction. In an early phase after ligand binding, SDS-stable CD95-aggregates interact with actin filaments to form large clusters (capping). These caps then internalize in a clathrin-dependent manner, forming CD95-receptosomes [30–32]. Inhibition of CD95 receptor internalization in type I cells results in a blockade of apoptosis and in induction of the ERK and NF-κB signaling pathways enlightening the crucial role of CD95 receptor compartmentalization for apoptosis induction [28, 33, 34].

The endolysosomal enzyme acid sphingomyelinase (A-SMase) is activated after CD95, TNF-R1 and TRAIL receptor stimulation and also plays an important role in various death signaling pathways [35–41]. A-SMase catalyses the hydrolysis of sphingomyelin to generate the pro-apoptotic second messenger ceramide. The enzyme can be activated by different mechanisms including posttranslational modifications and proteolytic cleavage [42–44]. We have shown that hydrolysis of phosphatidylcholine by phosphatidylcholine-specific phospholipase C results in the formation of diacylglycerol, activating protein kinase C (PKC) and A-SMase [45]. Furthermore, it was demonstrated that PKCδ phosphorylates A-SMase at Ser508, resulting in its activation and translocation to the plasma membrane [46]. Reactive oxygen species (ROS) have also been described to activate A-SMase possibly by inducing dimerization of the protein [47–50]. Moreover, we recently have shown that stimulation of TNF-R1 leads to a sequential activation of caspase-8 and caspase-7 resulting in proteolytic cleavage of A-SMase to yield higher enzymatic activity [51].

Studies revealed that after binding of CD95L or agonistic APO-1 antibody, small amounts of FADD and caspase-8 are recruited to the receptor, leading to the activation and translocation of A-SMase to the plasma membrane [52, 53]. In this context it has been reported that translocation of A-SMase is a Syntaxin-4 dependent process [55]. Cell surface A-SMase generates ceramide which in turn forms membrane platforms resulting in the clustering of CD95 receptors. These clusters are necessary for the internalization of the receptor and promote CD95 death signaling [56, 57]. The importance of A-SMase in the signaling cascade was demonstrated by inhibition or genetic deficiency of A-SMase which blocked the pro apoptotic CD95 signaling [38, 39, 58, 59].

So far, contribution of A-SMase to CD95 signaling has only been described for the first minutes after engagement while its importance at later time points is still unknown. Here, we investigated A-SMase activity at early and late stages of CD95 signaling to determine the localization and the role of A-SMase for the induction of apoptosis. We showed that the physiologically active CD95L induced A-SMase activation not only within the first minutes, but also at later time points in a second phase starting 30 minutes after CD95 stimulation. Immunofluorescence analyses and CD95-receptosome isolation indicated that A-SMase is located in such vesicles and is activated in a biphasic manner. The caspase-8 inhibitor IETD blocked the first activation peak suggesting an initial caspase-8-dependent activation of A-SMase. The second phase could be blocked with the dynamin inhibitor Dynasore indicating an internalization-dependent A-SMase activation mechanism. The caspase-3/−7 inhibitor DEVD also blocked the second phase of activation. In immunofluorescence analyses, we observed a co-localization of internalized CD95 with caspase-3 or -7 and their active forms. Immunoprecipitation of A-SMase revealed a direct interaction of pro-caspase-3 and -7 and the cleaved and activated caspase-7.

These present results suggest a so far unknown signaling pathway where internalized CD95-receptosomes fuse with trans-Golgi vesicles containing A-SMase, which in turn gets activated by caspases-3/−7 within these multivesicular bodies.

## RESULTS

### Soluble CD95L induces caspase dependent cell death and biphasic A-SMase induction in SKW6.4 cells

**Figure 1:**
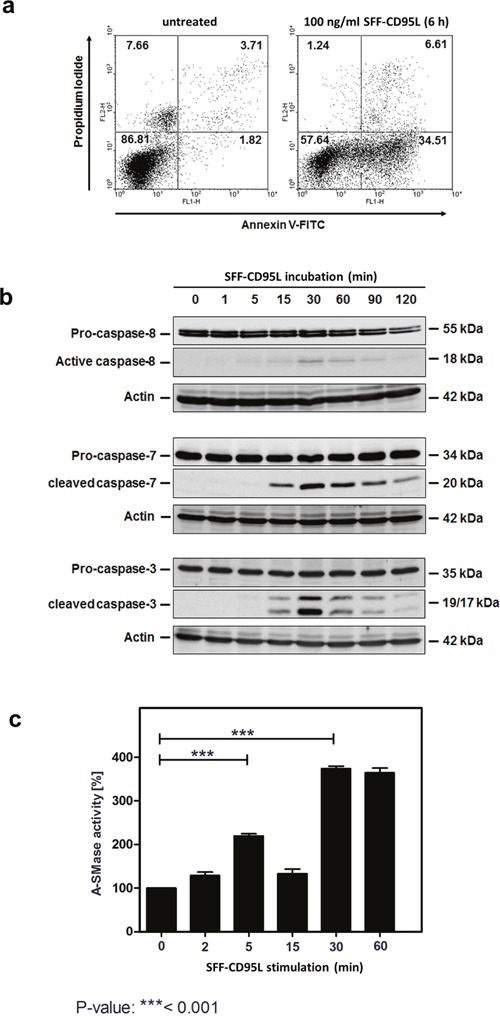
Apoptosis inducing SFF-CD95L activates the A-SMase in SKW6.4 cell lysates in a biphasic manner **a**. Induction of apoptosis in SKW6.4 cells after treatment with 100 ng/ml SFF-CD95L analyzed by AnnexinV/propidium iodide staining. **b**. Western blot analysis of SFF-CD95L induced activation of caspases. Initiator caspase-8 as well as effector caspases-3 or -7 showed a type I cell line dependent activation kinetic. **c**. Time course of A-SMase activity determined in SKW6.4 cell lysates after SFF-CD95L treatment. The activity maximums appeared after 15 and 90 minutes SFF- CD95L stimulation (n=3, 1way-Anova with Bonferroni's multiple comparison test).

We investigated the biological outcome of soluble CD95 ligand (CD95L) treatment of SKW6.4 cells. In this study we used a CD95L which was N-terminally modified with an Fc-, Strep- and a FLAG-tag (SFF-CD95L). Figure 1a shows the apoptosis induction after treating cells for 6 hours with SFF-CD95L measured by FACS: After incubation, an increase in Annexin V positive cells from 1.8 to 34.5% was measured and PI positive cells increased from 3.7 to 6.6%.

Figure 1b shows the time course of CD95L induced activation of the caspases-8, -7 and -3 in Western blots of total cell lysates.

In earlier studies, we have demonstrated that apoptosis induction in type I tumor cells depends on the induction of the ‘lysosomal-mitochondrial apoptosis amplification loop’ [28, 33]. We recently showed that in TNF-induced apoptosis, the sequential activation of caspases-8 and -7 lead to the activation of the lysosomal enzyme A-SMase. Interestingly, when we now treated cells with CD95L we recorded a bi-phasic activation of A-SMase in respective cell lysates (Figure 1c). The first activation peak occurred at 5 min of CD95L-treatment, whereas the second activation phase started after 30 min.

### Soluble CD95L induces CD95 internalization in type I cells

**Figure 2:**
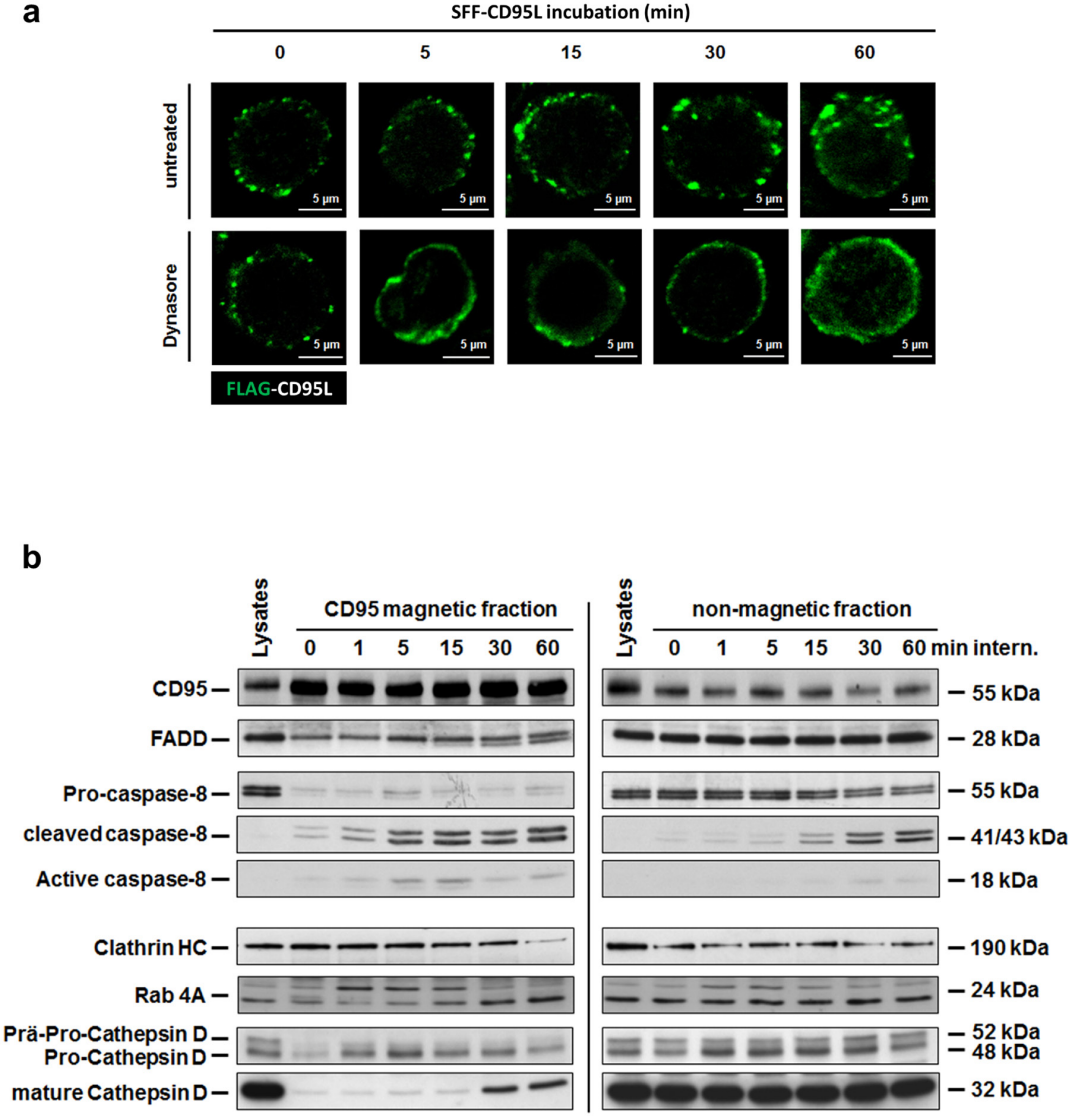
The SFF-CD95L induces the CD95-internalization in type I cells **a**. Immunofluorescence analysis of SFF-CD95L induced CD95 internalization in HuT78 cells. The CD95L induced a time-dependent internalization of the CD95 receptor which is blocked by the dynamin-specific inhibitor Dynasore. **b**. Time course of intracellular CD95-receptosome trafficking (left panel) and corresponding non-magnetic fractions (right panel) in SKW6.4 cells. Magnetic and non-magnetic fractions derived after indicated times of CD95 internalization were analyzed for CD95, DISC proteins (FADD and Caspase-8), clathrin (Clathrin HC), early endosomes (Rab 4A) and lysosomes (Cathepsin D).

In earlier studies on CD95-signaling in type I tumor cells and also in our previous studies on TNF-signaling, we found that ligation of the receptor resulted in their internalization [33, 34, 51, 60, 61]. As shown by fluorescence microscopy in Figure 2a, ligand binding also induced CD95-internalization in HuT78 cells (upper panel). Pre-treatment of the cells with the dynamin inhibitor Dynasore blocked CD95-internalization (lower panel). To support these findings, we magnetically isolated CD95-receptosomes from CD95L-treated SKW6.4 cells after different time points of internalization. These type I cells have been selected instead of Hut78 cells because of the higher yield of receptosomes material. The results are shown in Figure 2b (left part): The first panel shows the enrichment of CD95 in the magnetic fractions (panel one). The following panels show the time dependent recruitment of the adaptor protein FADD (panel two) and caspase-8 as well as its activation within receptosomes (panels four and five). To follow the intracellular trafficking of CD95-receptosomes, we analyzed the recruitment of clathrin and Rab4 (panels six and seven). The recruitment and activation of cathepsin D (panels eight and nine) confirmed our earlier findings of a fusion process between CD95-receptosomes with a lysosomal compartment, triggering the induction of the ‘lysosomal-mitochondrial apoptosis amplification loop’. The corresponding non-magnetic fractions of these preparations are shown in the right panel.

### A-SMase is activated in CD95-receptosomes

**Figure 3:**
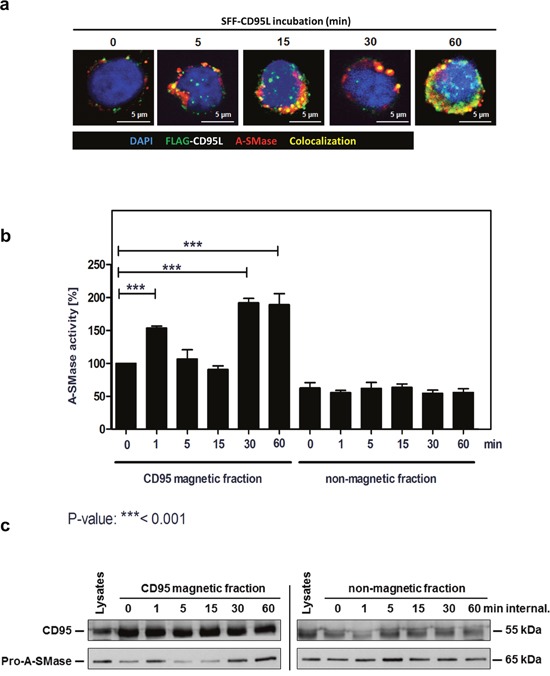
Biphasic activation of A-SMase in CD95-receptosomes **a**. Merged confocal microscopic images of SKW6.4 cells stimulated with 100 ng/ml SFF-CD95L. The CD95L was visualized by an anti-FLAG antibody and anti-mouse Alexa-Fluor 488. A-SMase stained by a polyclonal anti-A-SMase antibody and anti-rabbit Alexa-Fluor 555 was detected in CD95-receptosomes between 1 min and 60 min internalization. Co-localization of CD95 and A-SMase is indicated in yellow. **b**. Magnetic and non-magnetic fractions were used to measure A-SMase activation in SKW6.4 cells after indicated time points of CD95 internalization. Three independent preparations were pooled and measured in triplicate. (1way-Anova with Bonferroni's multiple comparison test) **c**. Western blot analysis of CD95 magnetic and non-magnetic fractions with anti-CD95 and anti-A-SMase antibodies. Corresponding to the activity assay, Western blot analysis demonstrated a biphasic appearance of A-SMase in the magnetic fractions.

To investigate the spatial distribution and a possible interaction of CD95 and A-SMase, we then analyzed the subcellular localization of CD95 and A-SMase by confocal laser-scanning microscopy (CLSM, Figure 3a). We observed a co-localization of both proteins already five minutes after ligand binding at the plasma membrane. Co-localization increased over time, indicating fusion events of CD95-receptosomes with lysosomes or A-SMase-carrying endosomes. To support these findings, we isolated CD95-receptosomes after various time points of internalization. Figure 3b (left panel) shows an initial activation of A-SMase already after 1 min of endocytosis initiation. The second activation phase occured 30 to 60 min after receptor-internalization and thus, is in perfect accordance with the lysosomal fusion observed by confocal laser-scanning microscopy (see Figure 3a). Of note, A-SMase activation was only observed in the CD95 magnetic fractions (receptosome preparations), but not in the non-magnetic residual lysate fractions, indicating that the activation of A-SMase is tightly linked to CD95.

The following Western blot analysis of CD95 and A-SMase in isolated CD95-receptosomes corroborated these findings (Figure 3c): A-SMase was recruited rapidly upon ligand binding (1 min) and 30 to 60 min after receptor internalization. Together, these findings clearly show that CD95 and A-SMase co-localize in two distinct subcellular compartments (plasmamembrane and endo-lysosomes) at different time points.

Based on studies of others and on our own findings for TNF-signaling, we assumed that the fission of newly formed receptosomes from the plasma membrane (PM) is finished at about 3-5 min after endocytosis induction [62, 63]. Thus, we believed that the early appearance of CD95/A-SMase co-localization was due to the translocation of A-SMase to the PM as already described by Grassmé and colleagues [52], while the later one required receptor endocytosis and fusion with a lysosomal compartment.

### The first phase: A-SMase activation early after ligand binding is caspase-8 dependent

**Figure 4:**
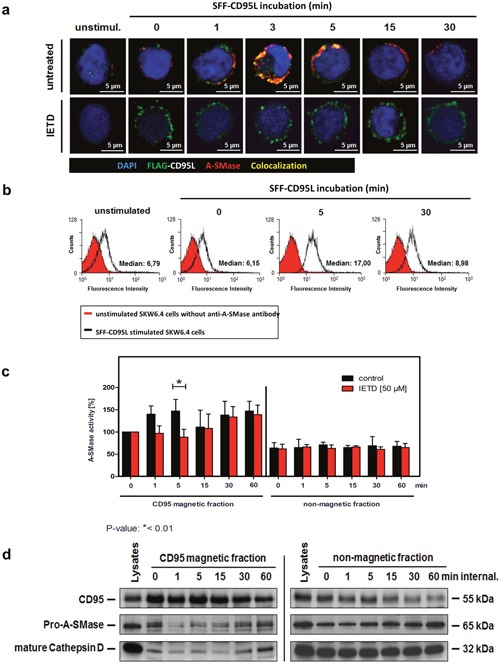
Characterization of the first A-SMase activation peak **a**. Translocation of A-SMase to the plasma membrane in non-permeabilized SKW6.4 cells. Stimulation with 100 ng/ml SFF-CD95L resulted in a rapid translocation of A-SMase to the cell surface (upper panel) which was abrogated after co-treatment with 50 μM caspases-8 inhibitor IETD (lower panel). **b**. Transient exposure of the A-SMase to the plasma membrane induced by SFF-CD95L. Non-permeabilized SKW6.4 cells were stimulated for indicated time points with 100 ng/ml SFF-CD95L and analyzed by flow cytometry. **c**. Comparison of A-SMase activation in CD95 magnetic and non-magnetic fractions from SKW6.4 cells treated with or without IETD (50 μM). In contrast to the control, IETD blocked the first activation peak of A-SMase in CD95 magnetic fractions. Represented are the mean values of four independently performed assays with control cells and three independent experiments with IETD treated cells. (2way Anova with Bonferroni post-test) **d**. Western blot analysis of CD95 magnetic and non-magnetic fractions isolated from IETD treated SKW6.4 cells with anti-CD95, anti-A-SMase and anti-Cathepsin D antibodies.

Previous reports suggested a role of caspase-8 in the internalization of CD95 upon ligand binding [31]. To analyse possible contribution of caspase-8 to the first phase of A-SMase activation, we treated cells with the caspase-8 inhibitor IETD before SFF- CD95L incubation or left them untreated and then compared CD95/A-SMase co-localization by CLSM. Figure 4a (upper panel) shows the co-localization of both molecules after 1 to 5 minutes. Pre-treatment with IETD prevented this co-localization (lower panel). In Figure 4b, we confirmed the CD95L triggered exposure of A-SMase to the plasma membrane with a maximum staining intensity after 5 min of ligand treatment. The analysis of A-SMase activation in isolated CD95-magnetic fractions confirmed these findings (Figure 4c): The biphasic activation of A-SMase is apparent in CD95L treated cells (left side, black columns). Caspase-8 inhibition by IETD blocked the first activation peak but still allowed later activation (red columns). A-SMase activation was not detected in the non-magnetic fractions (right panel). In the same magnetic and non-magnetic fractions, we analyzed the recruitment of pro-A-SMase and mature cathepsin D as a marker for the lysosomal compartment by Western blotting. Indeed, A-SMase recruitment could not be observed at the early time points (Figure 4d). Together, these findings implicate a crucial role for caspase-8 in the early A-SMase activation phase.

### The second phase: Late A-SMase activation requires CD95 internalization

**Figure 5:**
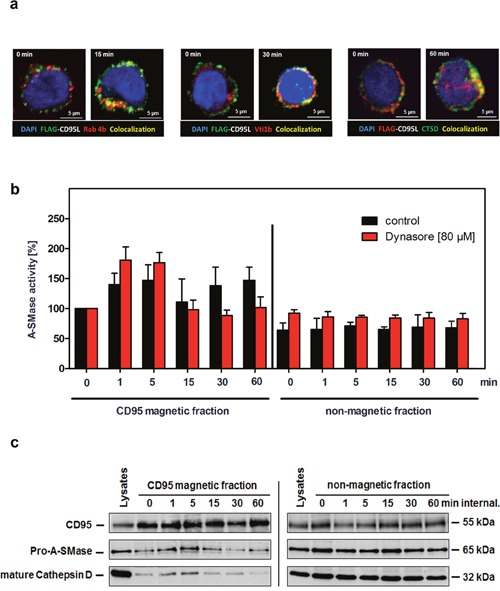
Characterization of the second A-SMase activation peak **a**. Immunofluorescence analysis of the intracellular CD95 trafficking in SKW6.4 cells. Localization analysis of SFF-CD95L/CD95 (green) with early endosomal marker Rab4A (red) or trans-Golgi marker Vti1b (red), and Cathepsin D. Co-localization is indicated in yellow. **b**. Measurement of A-SMase activation in control or Dynasore treated (80 μM) CD95 magnetic and non-magnetic fractions. The second activation peak of A-SMase in CD95 magnetic fractions was blocked by the dynamin inhibitor Dynasore. Represented are the mean values of four independently performed assays with control cells and two independent experiments with Dynasore treated cells. (n=2) **c**. Time course of CD95 internalization in magnetic and non-magnetic fractions isolated from Dynasore treated SKW6.4 cells. The fractions were analyzed with anti-CD95, anti-A-SMase and anti-Cathepsin D antibodies.

We next analyzed the intracellular CD95-trafficking by confocal laser-scanning microscopy in more detail. As detailed in Figure 5a, co-localization of CD95 with the early endosomal marker Rab4 was visible after15 min of receptor internalization. The intracellular maturation and fusion with trans-Golgi vesicles became apparent by the recruitment of Vti1b after 30 min (middle panel) and of Cathepsin D (CTSD) after 60 min (right panel) of CD95 internalization.

We already described the potency of the dynamin inhibitor Dynasore to prevent CD95 internalization (Figure 2a). We isolated CD95 magnetic fractions from Dynasore treated SKW6.4 cells after different time points. Measurement of A-SMase activity in these preparations revealed that the first activation still occurred while the second phase was absent (Figure 5b, left panel, red columns). In contrast, isolation of receptosomes from non-Dynasore treated cells showed both activation phases (black columns). Again, no A-SMase activation was observed in the non-magnetic fractions. We additionally analyzed CD95-magnetic fractions from Dynasore treated cells by Western blot probing for the recruitment of pro-A-SMase and CTSD: Both molecules were only recruited early after ligand binding. These findings indicate that the second A-SMase activation phase depended on receptor internalization and the subsequent fusion of receptosomes with the lysosomal compartment.

### A-SMase activation within CD95-receptosomes requires caspase-3 or -7

**Figure 6:**
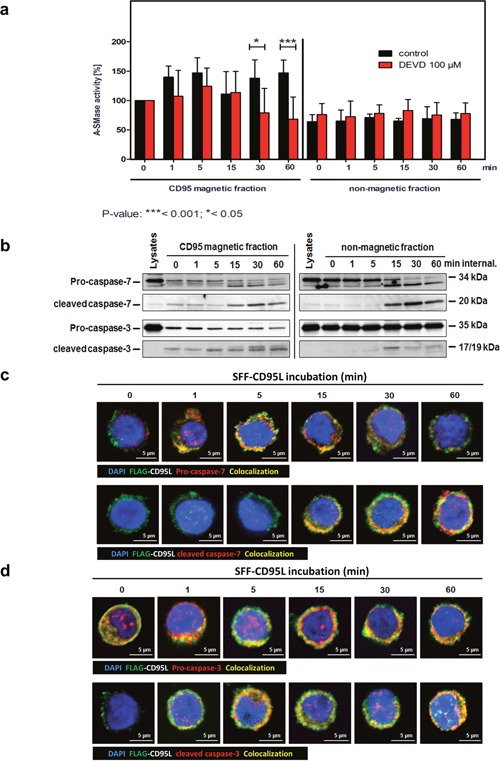
Caspase-3 or -7 are responsible for the second A-SMase activation peak in CD95-receptosomes **a**. A-SMase activity in magnetic and non-magnetic fractions isolated from control or DEVD (inhibitor of caspase-7 / -3) treated SKW6.4 cells. DEVD decreases the second activation peak of A-SMase in the magnetic fraction demonstrating the potential role of caspase-7 and -3 in the activation of the A-SMase. Represented are the mean values of four independently performed assays with control or DEVD treated cells (2way Anova with Bonferroni post-test). **b**. Western blot analysis of CD95 magnetic and non-magnetic fractions isolated from SKW6.4 cells with anti-pro-caspase-7, anti-cleaved-caspase-7, anti-pro-caspase-3 and anti-cleaved-caspase-3 antibodies. **c** and **d**. Merged confocal microscopic images of SKW6.4 cells shows a co-localization (yellow) between SFF-CD95L/CD95 (green) and pro-caspase-7, cleaved-caspase-7, pro-caspase-3 or cleaved-caspase-3 (red) at indicated times of CD95 internalization.

In an earlier study, we described the requirement of caspase-7 for A-SMase activation in response to TNF [51]. Based on these observations, we next pre-treated SKW6.4 with the caspase-3/−7 inhibitor DEVD and subsequently isolated CD95-receptosomes (Figure 6a). A-SMase activity assays of these fractions revealed that its activation was blocked in the inhibitor treated cells (red columns, left panel). In untreated cells, the previously described biphasic activation was apparent (black columns). Western blot analyses of CD95 magnetic fractions revealed the recruitment and activation of both caspase-3 and -7 to CD95-receptosomes at late time points (Figure 6b).

Subsequent to the biochemical analysis of CD95 magnetic fractions we performed localization analyses by confocal laser-scanning microscopy. Figure 6c documents co-localization of both, pro-caspase-7 (upper panel) and cleaved caspase-7 (lower panel) with SFF-CD95L bound to CD95. The cleavage/activation of caspase-7 appeared clearly following (15-60 min) its initial recruitment. We observed a similar co-localization of CD95 with both, pro- and cleaved caspase-3 (Figure 6d). These observations indicated that the CD95 induced A-SMase activation depended on the enzymatic activity of caspase-3 or -7 and that both enzymes were recruited and activated in internalized CD95-receptosomes.

### A-SMase interacts with caspase-3 or -7

**Figure 7:**
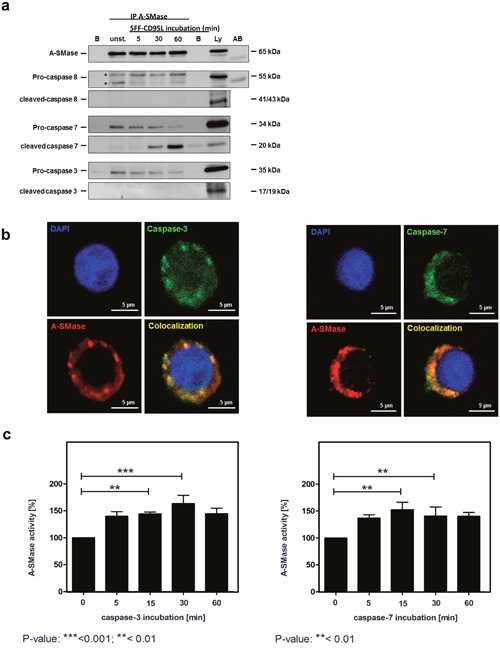
A-SMase interacts with caspases-3 or caspases-7 **a**. Immunoprecipitation of A-SMase with an anti-A-SMase antibody from unstimulated and SFF-CD95L stimulated HeLa wild-type cell lysates. Western blots show that pro-caspase-3, pro-caspase-7 and cleaved-caspase-7 co-precipitates with A-SMase. **b**. Immunofluorescence analysis visualize a co-localization (yellow) between A-SMase (red) and caspases-3 or caspases-7 (green) in unstimulated SKW6.4 cells. **c**. Activation of immunoprecipitated A-SMase-GFP after 15 min incubation with caspase-3 or caspase-7 (n=3, 1way Anova with Bonferroni multiple comparison test).

To investigate a physical interaction between A-SMase and Caspase-3 or -7, we performed immunoprecipitation assays of A-SMase and analyzed co-precipitation with caspase-8, -7 and -3 (Figure 7a). For these experiments, we used stably eGFP-A-SMase overexpressing HeLa cells [51]. eGFP-A-SMase could be precipitated from untreated and CD95L-treated cell lysates (first panel). Pro-caspase-8 was constitutively co-precipitated but not the active form (second and third panel). Both, pro-caspase-7 (middle panel) and pro-caspase-3 (lower panel) were co-precipitated. For caspase-7, also the cleaved fragment was co-precipitated (middle panel). The constitutive interaction between A-SMase and caspase-3 and -7 was substantiated by co-localization analysis by confocal laser-scanning microscopy of non-stimulated SKW6.4 cells (Figure 7b).

In order to address the question of a functional relevance of the CD95 association with A-SMase, we treated immunoprecipitated A-SMase preparations either with recombinant active caspase-3 (Figure 7c, upper panel) or -7 (lower panel) or left them untreated. Subsequent measurement of A-SMase activity revealed that both enzymes induced A-SMase activation with a similar potency.

All in all the present results demonstrate that A-SMase and both caspases-3 and -7 co-localize in the same CD95-containing intracellular compartment, physically interact with each other and that both caspases can directly stimulate A-SMase activity.

## DISCUSSION

A-SMase plays an important role in death-receptor mediated apoptosis. For TNF-R1 signaling, we have shown that A-SMase is cleaved and activated via a sequential proteolytic activation of caspase-8 and caspase-7 within TNF-R1 receptosomes [51]. For CD95 signaling it was described that A-SMase is activated and translocates to the plasma membrane forming ceramide-enriched membrane platforms. Since the role of intracellular A-SMase after CD95 internalization had not been investigated, we analyzed the molecular mechanism in more detail.

Most studies on CD95 signaling have been performed with the agonistic antibodies APO1-3 or CH11 [28, 40, 64, 65]. In the present work we used a CD95L which was N-terminally modified with an Fc-, Strep- and a FLAG-tag. The human IgG1 Fc region allowed the oligomerization of CD95L which is necessary for the induction of apoptosis [19]. The biological activity of the purified ligand was measured by the exposure of Annexin V on the cell surface. After 6 h of incubation, more than 30% of the cells were already Annexin V positive. In line with this, the cleavage of caspase-8, -3 and -7 further demonstrated the SFF-CD95L-induced apoptosis. The biological activity of physiological CD95L has already been described before, however, it was still unclear if this ligand could induce internalization of CD95 [33, 66]. For this, all further analyses were performed with SFF- CD95L to answer this question.

Immunofluorescence analysis and Western blot confirmed the internalization of the CD95 after binding of CD95L. These results are in contrast to data obtained by Chaigne-Delalande and colleagues who published that internalization of CD95 was just detectable after stimulation with the agonistic antibody APO1-3 whereas the modified CD95L did not induce internalization of the receptor [64]. On the other hand, Lee and colleagues as well as Eramo and colleagues demonstrated that CD95L is able to induce CD95 receptor internalization [33, 66]. In the present work the CD95 internalization was confirmed by immunofluorescence analysis using the inhibitor Dynasore which blocks the fission of endocytotic vesicles. While the CD95L-CD95 receptor complex was internalized upon temperature shift to 37°C, Dynasore blocked this process resulting predominantly in cell surface localization of CD95. CD95 receptor internalization was described to be an actin and clathrin-dependent process [34].

Grassmé and colleagues demonstrated that an early activation of A-SMase is involved in CD95 clustering [52, 57]. In addition to early A-SMase activation, we showed a second activation phase occurring at 30 to 60 min in SFF-CD95L stimulated SKW6.4 lysates. The observed effects in lysates were confirmed by isolation of CD95-receptosomes and measurement of A-SMase activity. Analyzing magnetic and non-magnetic fractions of CD95 receptosomes preparations, we were able to show that A-SMase is activated in a biphasic manner in magnetic fractions whereas activity in non-magnetic fraction remained unchanged. Western blot analysis confirmed the biphasic appearance of A-SMase in the magnetic fractions. Immunofluorescence analysis of CD95L and A-SMase demonstrated that co-localization of both proteins was detectable already after 5 min up to 60 min. The first activation peak has already been described, suggesting that caspase-8 is involved in the activation process [52–54].

Using the caspase-8 inhibitor IETD, we were able to show that the first activation peak is caspase-8 dependent. By CLSM analysis, A-SMase activity assays and Western blot analysis, we demonstrated that A-SMase translocates to the plasma membrane and that activation after early time points was blocked whereas the later activation peak was still present. These results support the finding by Grassmé and colleagues, that translocation and activation of A-SMase at the plasma membrane is a caspase-8-dependent process [52]. Perrotta and colleagues demonstrated that Syntaxin-4 is also involved in the translocation and activation of A-SMase after CD95 stimulation. The translocation of A-SMase is associated with a fusion process of A-SMase positive lysosomes with the plasma membrane mediated by Syntaxin-4 [55]. Blocking Syntaxin-4 could be a further target to inhibit A-SMase activation, ceramide-enriched platform formation and CD95 receptor internalization. Although the first activation peak could be blocked by IETD, the second activation peak remained unchanged, indicating a caspase-8-independent activation mechanism in that case.

Western blot analysis of magnetic fractions demonstrated the biphasic appearance of A-SMase in CD95-receptosomes correlating with an increased A-SMase activity. Since no increase of A-SMase activity was measured in non-magnetic fractions, we hypothesized that A-SMase is incorporated into to CD95-receptosomes either by co-internalization from the plasma membrane or by fusion processes of trans-Golgi vesicles with internalized CD95 containing vesicles, similar as described by us for the TNF-R1 system [43]. The results obtained with the caspase-8 specific inhibitor IETD demonstrated a block of the first activation peak and an inhibition of the translocation of A-SMase. However, the second peak remained unchanged under these conditions, indicating that co-internalization does not play a role, since the second activation peak should have been blocked as well. Fusion processes with intracellular vesicles were already described for TNF-R1 [61, 62]. Pre-pro-CTSD and A-SMase carrying trans-Golgi vesicles fuse with the internalized TNF-R1 forming multivesicular bodies. During the fusion and maturation process marker proteins for early endosomes (Rab5), trans-Golgi vesicles (Vti1b) and late endosomes (Lamp-1) were found to co-localize with TNF-R1 [62].

The Western blot analysis of the magnetic and non-magnetic fractions demonstrated, that upon CD95L binding, proteins of the DISC (FADD and caspase-8) were recruited and activated. Fusion of CD95-receptosomes with intracellular vesicles could be observed by increased amount of the endosomal marker protein Rab4A at 1 to 15 min and endo-lysosomal protein CTSD at 30 and 60 min of internalization. Confocal immunofluorescence analysis demonstrated a co-localization of CD95 receptor with Rab4A (15 min), Vti1b (30 min) and CTSD (60 min). These findings confirm the results of Lee and colleagues and demonstrate that CD95L induces the CD95 internalization. Similar to the TNF-R1 system, CD95 receptosomes fuse with intracellular vesicles and form multivesicular bodies [33, 62].

Because A-SMase is present and activated in CD95 receptosomes, we analyzed if internalization is required for the second activation peak. Therefore, Dynasore was used to block the internalization of the receptor. Isolation and analysis of CD95 magnetic fractions revealed, that A-SMase is not activated at the second peak in comparison to untreated control cells. Western blot analysis confirmed that the amount of A-SMase is clearly reduced at 30 and 60 minutes of CD95 activation. Studies on internalization blockade demonstrated that cells are protected from cell death [33, 34]. CD95 receptors which were retained at the cell surface induce pro-survival and proliferative signaling cascades by activation of ERK and NF-κB. In contrast, CD95 internalization leads to a massive recruitment of DISC components to the receptor and activation of caspases inducing apoptosis [33, 34].

According to the obtained results, the first activation peak correlated with caspase-8 and the second activation peak with receptor internalization. However, the precise mechanism remains enigmatic. A-SMase can be activated by different mechanisms including phosphorylation, ROS or proteolytic cleavage [46–48, 51]. Based on mechanistic similarities between TNF-R1 and CD95 signaling, especially with regard to receptor internalization, we observed further parallels. According to Edelmann and colleagues, A-SMase is cleaved and activated by caspase-7 [51]. To test if other caspases than caspase-8 are involved in the second activation peak, we used the caspase-3/−7 inhibitor DEVD. CD95-receptosomes isolated form DEVD treated SKW6.4 cells did not show any A-SMase activity at late but activation at early time points. Western blot analysis of CD95 magnetic fractions revealed the presence of pro-caspase-3/−7 as well as active caspase-3/−7 in CD95-receptosomes. These findings were confirmed by immunofluorescence analysis showing a co-localization between the ligand-receptor complex and caspase-3 or -7 and active caspases-3 or -7, respectively. A possible activation mechanism of caspase-3 and -7 could be mediated by CTSD. Conus and colleagues demonstrated that CTSD is able to cleave and activate inactive caspase-8 at acidic pH leading to homodimerisation of the caspase and activation of a proteolytic cascade [67]. A similar mechanism might be possible for A-SMase activation in CD95-receptosomes. Activated caspase-8 leads to a proteolytic cleavage of caspase-3 and -7 which in turn activates the A-SMase. This hypothesis was supported by the detection of the active form of CTSD in CD95 receptosomes. Calpain, a Ca^2+^-dependent cysteine-protease, could be a further candidate for caspase-7 activation. It was shown, that calpain activates caspase-7 in a caspase-8 independent manner by depolarization of mitochondria and the release of cytochrome c [68]. This mechanism could replace caspase-8 for activation of caspase-7 within the CD95 receptosomes and would support our observation that blocking of caspase-8 does not prevent the second activation peak of A-SMase.

How does caspase-3 and -7 enter CD95-receptosomes? For caspase-3 it was demonstrated that it is located in lipid rafts at the plasma membrane [69]. Furthermore, the authors showed that caspase-3 is a component of the CD95-DISC and is necessary for a complete activation of caspase-8. These observations support the idea that caspase-3 is co-internalized with CD95. We revealed the presence of pro-caspase-3 and active caspase-3 in magnetic fractions at all time points of internalization, confirming a possible co-internalization. In contrast to caspase-3, caspase-7 was only detected after 15 to 60 min after internalization. This late appearance of caspase-7, points to different mechanisms of incorporation compared to caspase-3. Two independent studies of CD95 demonstrated that upon stimulation, active caspase-7 was detected in microsomal fractions [70, 71]. Since microsomal fractions are a mixture of vesicles derived from the endoplasmic reticulum, Golgi apparatus, plasma membrane and lysosomes, the obtained results support the association of caspase-7 with vesicles and membranes. Therefore, one probable mechanism would be the fusion of vesicles with the internalized CD95-receptosomes, leading to incorporation of caspase-7 in these compartments. By fusion of vesicles, multivesicular compartments are formed where caspase-3 and -7 are separated from A-SMase by a membrane. Slow acidification of the vesicles can lead to the disruption of the limiting membranes enabling interaction between the proteins. Caspase-7 is enzymatically active between a pH of 6.5 and 7.7 and caspase-3 between a pH of 6.4 and 8.6 [51, 72]. This indicates that the acidification in the multivesicular compartments does not immediately block caspase activity and allows caspase-3 or -7 to cleave and activate A-SMase.

In order to verify an interaction between A-SMase and the effector-caspases-3 or -7, co-immunoprecipitation and immunofluorescence analyses were performed. Both methods demonstrated a constitutive interaction between A-SMase and caspase-3 or -7 in unstimulated cells. A similar finding had been reported by Matsumoto et al, showing a nitric oxide-dependent interaction of A-SMase and pro-caspase-3. However, this group speculated on a mechanism in which A-SMase protects pro-caspase-3 from initiator-caspases induced cleavage [73]. This could explain the missing signal for cleaved-caspase-3 in the co-immunoprecipitations. In case of the interaction between A-SMase and caspase-7 comparable results were obtained in our previous report [51]. However, the present findings also demonstrate an interaction between A-SMase and cleaved-caspase-7 in CD95L-stimulated cells, suggesting the potential role of caspase-7 for activation of A-SMase. These results were supported by the *in vitro* experiments where exogenous caspase-7 as well as caspase-3 activated precipitated A-SMase-GFP.

**Figure 8:**
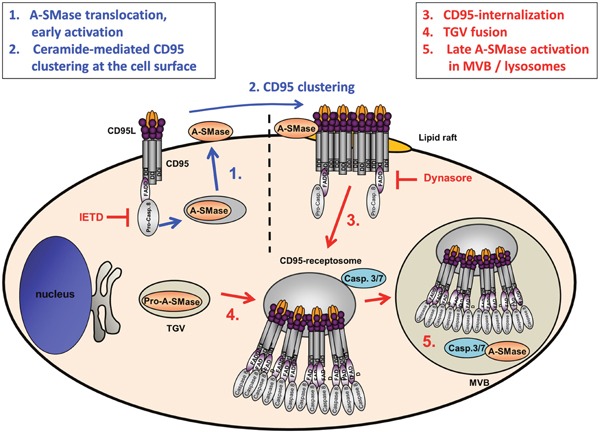
Model of CD95L induced A-SMase activation Biphasic activation of A-SMase in CD95-receptosomes is caused by two different mechanisms. CD95 ligation leads to the activation of caspase-8 which triggers a translocation of A-SMase onto the outer leaflet of the plasma membrane. At the plasmamembrane A-SMase colocalizes with CD95 and is supposedly involved in the formation of lipid rafts propagating the formation of CD95 clusters [52]. In type I cells, these receptor ligand complexes undergo clathrin-dependent internalization forming CD95-receptosomes. Along the endocytotic pathway CD95-receptosomes fuse with trans-Golgi vesicles (TGV) which contain A-SMase to form multivesicular bodies (MVB) which eventually mature to early lysosomes. Within this compartment, caspase-7 or caspase-3 activates A-SMase to transmit further downstream signaling.

The results described above are schematically summarized in Figure 8. In conclusion, the present findings demonstrated a CD95L-induced biphasic activation of A-SMase. The earlier phase is based on the A-SMase translocation to the cell surface and might be involved in receptor endocytosis. The latter activation is based on CD95-receptosome/endosome/lysosome fusion events and is probably involved in the lysosomal-mitochondrial apoptosis induction.

## MATERIALS AND METHODS

### Chemicals and inhibitors

Dynasore was obtained from Sigma Aldrich (Germany), caspase 3/7 inhibitor Z-Asp(OMe)-Glu(OMe)-Val-DL-Asp(OMe)-fluoromethylketone (Z-DEVD-FMK) and caspase-8 inhibitor Z-Ile-Glu(OMe)-Thr-DL-Asp(OMe)-fluoromethylketone (Z-IETD-FMK) were obtained from Bachem (Switzerland). The Apoptosis (Annexin V/propidium iodide) kit was obtained from Roche and protein G microbeads were obtained from Miltenyi Biotech. Exogenous caspase-3 and -7 were obtained from Biomol (Germany).

### Antibodies

The goat anti-actin antibody (C11), mouse anti-FAS (CD95) antibody (C20) and rabbit anti-Rab4A antibody (D20), mouse anti-caspase-3 antibody (E8), mouse anti-caspase-7 antibody (CSP03) were obtained from Santa Cruz Biotechnology. Rabbit anti-caspase-7 antibody (E22), rabbit anti-caspase-3 (E61), rabbit anti-caspase-8 antibody (E7), mouse anti-CTSD antibody (CTD-19), rabbit anti-A-SMase antibody (ab83354) and mouse anti-A-SMase antibody (ab74281) were obtained from Abcam. Rabbit anti-cleaved caspase-8 antibody (18C8), rabbit anti-caspase-3 antibody (8G10), rabbit anti-cleaved caspase-3 antibody (9661) and rabbit anti-cleaved caspase-7 antibody (9491), rabbit anti-clathrin heavy chain (D3C6), rabbit anti-A-SMase antibody (3687) and rabbit anti-FADD antibody (2782) were obtained from Cell Signaling. The mouse anti-M2-Flag antibody (F1804) and rabbit anti-Flag (SIG1-25) were obtained from Sigma Aldrich. Rabbit anti-Vti1b (164002) was obtained from Synaptic Systems, rabbit anti-GFP antibody (A11122) was obtained from Invitrogen and HRP-conjugated mouse anti-GFP was obtained from Miltenyi Biotech. Rabbit polyclonal anti-A-SMase antibody was generated by Areta International s.r.l. (Gerenzano, Italy). The secondary antibodies Alexa Fluor 488 labelled anti-mouse IgG antibody (A21202), Alexa Fluor 555 labelled anti-mouse IgG antibody (A21422) and the Alexa Fluor 555 labelled anti-rabbit IgG antibody (A31572) were obtained from Invitrogen/Molecular Probes. HRP conjugated donkey anti-goat antibody (705-035-003), HRP conjugated rabbit anti-mouse antibody (315-035-045) and HRP conjugated goat anti-rabbit antibody (111-035-045) were from Dianova and HRP conjugated mouse anti-rabbit light chain antibody (MAB201P) was obtained from Millipore.

### Cell culture

Human SKW6.4, HuT78, HeLa and HEK293 were purchased from ATCC. HeLa cells stably overexpressing EGFP-A-SMase were described before. HeLa, MEF and HEK 293T cells were maintained in DMEM+HEPES culture medium (Life Technologies) and HuT78 and SKW6.4 cells were maintained in RPMI 1640 medium (Life Technologies). Both media were supplemented with 10% fetal calf serum, 10 mM glutamine, and 0.1 mg/ml gentamycin.

### Expression and purification of CD95 ligand (CD95L)

HEK 293T cells were transfected with a plasmid coding for Strep-, Fc- and FLAG-tagged CD95L (SFF-CD95L), by electroporation, transferred into Gibco ®FreeStyle ^TM^ 293 medium and cultivated for 2 days. The supernatant was collected and cells were again incubated for 2 days adding 1 mg/ml G418 (Biochrom). The collected supernatants were applied onto a protein G column (GE Healthcare), washed with 20 mM Na_2_PO_4_ pH7,0 and the bound protein was eluted using 0.1 M glycine pH 2.7. The eluate was neutralized using 1 M Tris-HCl pH 9.0. Purity and activity of the CD95L was tested by Western blotting and apoptosis assay.

### Preparation of cell lysates

Cells were lysed with homogenisation buffer (40 mM HEPES pH 7.4; 150 mM KCl; 0.2% NP40) by freezing and thawing of the samples. The cells were resuspended using a syringe and a 21G needle. The lysate was centrifuged for 10 min at 20,000 x g and 4°C. The supernatant was used for BCA protein determination.

### Immunoprecipitation

Immunoprecipitation of endogenous A-SMase, caspase-3, caspase-7 or cleaved caspase-7 was performed using 1,8 × 10^7^ HeLa cells cooled on ice for 30 min followed by incubation of 100 ng/mL CD95L for 1 hour on ice. Temperature shift to 37°C for indicated time points was stopped by addition of 1 mL ice cold PBS and immediately centrifugation at 300 x g for 5 min at 4°C. Cell lysates were prepared as described above. Protein G coupled antibodies were added and the mixture was incubated under rotation for 1 hour at 4°C. Lysate was loaded onto a pre-equilibrated μMacs column (Miltenyi Biotech), washed with homogenization buffer and the precipitated protein was eluted according to the manufacturers description.

Immunoprecipitation of EGFP-tagged A-SMase was performed as previously described (Edelmann *et al*, 2011) followed by incubation with 50 units of exogenous caspase-3 or caspase-7 at 37°C for 15 min. 10 μl of the reaction mixture was used for A-SMase activity determination.

### A-SMase activity assay

Activity of A-SMase in cell lysates, CD95-receptosomes or immunoprecipitated protein was measured using N-methyl-[14C]-sphingomyelin (0.5 μCi/ml, 0.55 Ci/mmol, Perkin Elmer) as substrate in 250 mM Na-acetate pH 5.0, 1 mM EDTA, 0.1% Triton X-100. 5-12 μg of protein was incubated with the substrate for 2 h at 37°C in a total volume of 150 μl. The reaction mixture was extracted using 750 μl chloroform/methanol (2:1) and 250 μl H_2_O. Radioactivity of enzymatically liberated radioactive phosphorylcholine in a 250μl aliquot of the aqueous phase was measured in a beta-counter.

### Western blot analysis

Cells were lysed as described above and proteins were separated on 10 to 15% SDS-PAGE. Immunoblotting was performed using one of the primary antibodies indicated above and secondary antibody horseradish peroxidase conjugates. Blots were developed using the ECL detection reagent (GE Healthcare).

### Preparation and analysis of magnetic CD95 containing membrane fractions

Pellets of 1.5 × 10^8^ cells were incubated on ice for 30 min followed by addition of 500 ng of Fc-tagged CD95L and 40 μl protein-G microbeads (Miltenyi Biotech). The reaction mixture was incubated for 1 hour on ice, washed twice with ice-cold PBS and pelleted by centrifugation at 100 x g for 10 min at 4°C. Formation of magnetically labelled CD95-receptosomes was achieved by incubation of cells at 37°C for various times as indicated in the figure legends. Subsequently, cells were homogenized by sonication in a 0.25 M sucrose buffer, supplemented with 15 mM HEPES pH 7.4, 0.5 mM MgCl_2_ and the Complete® protease inhibitor cocktail (Roche) at 4°C. A post-nuclear supernatant was applied for magnetic separation of CD95-receptosomes in a high-gradient magnetic field generated in a custom-built free-flow magnetic chamber (Tchikov and Schütze, 2008). Magnetic fractions were separated by SDS-PAGE and analyzed by Western blot, or were used directly for A-SMase activity assays.

### Translocation experiments

1 × 10^6^ cells were incubated in serum-free medium for 30 min on ice followed by 1 h incubation with 100 ng/ml CD95L. Temperature shift was performed according to the figure legend and stopped using ice-cold RPMI medium. Cells were incubated with anti-A-SMase antibody (Areta International s.r.l. (Gerenzano, Italy) for 1 h on ice, washed and incubated with the Alexa Fluor 555 conjugated anti-rabbit antibody for 1 h. After washing with PBS, translocation of A-SMase was analyzed using FACS-Calibur (BD) and Cell-Quest 3.3 software.

### Apoptosis assay

5 × 10^5^ cells were seeded in six-well plates; pretreated with or without 80 μM Dynasore for 30 min and stimulated with 100 ng/ml CD95L for 6 hours. The cells were harvested and washed once with PBS and centrifuged 5 min at 200 x g. The cell pellet was resuspended in Annexin-V-FLOUS labelling solution containing Annexin V and propidium iodide (PI) (according to the manufacturer's instructions) and incubated for 10-15 min at room temperature. Stained cells were analyzed using the FACS-Calibur (BD) and the Cell-Quest 3.3 software.

### Confocal microscopy

5 × 10^5^ cells were incubated for 30 min at 37°C on adhesions slides (Squarix, Germany) with or without the inhibitors Dynasore (80 μM) or IETD (50 μM) followed by 30 min incubation on ice. When incubating the cells with inhibitors, all following steps were performed in the present of the inhibitor. Adherent cells were incubated with 100 ng/ml CD95L for 1 h on ice, washed with PBS and incubated at 37°C as indicated in the figure legends. Internalization was blocked by washing once with PBS followed by fixation of the cells with 4% PFA for 30 min. Cells were permeabilised with PBS supplemented with 0.2% BSA and 0.1% Saponin. Co-localization of CD95L with A-SMase, CTSD; Vti1b, Rab4A, caspase-3, cleaved caspase-3, caspase-7 and cleaved caspase-7, respectively was analyzed. Anti-M2 Flag antibody (mouse or rabbit) in combination with the other primary antibodies (A-SMase, CTSD, Vti1b, Rab4A, caspase-3, cleaved caspase-3, caspase-7 and cleaved caspase-7) were incubated for 1 h at RT followed by secondary Alexa Fluor 488 conjugated anti-mouse secondary antibody and Alexa Fluor 555 conjugated anti-rabbit secondary antibody as indicated in the figure legends.

Co-localization of A-SMase and caspase-3 or caspase-7 was analyzed by incubation of rabbit anti-A-SMase antibody with mouse-anti-caspase-3 or caspase-7 antibody followed by incubation of secondary Alexa Fluor 488 conjugated anti-mouse secondary antibody and Alexa Fluor 555 conjugated anti-rabbit secondary antibody.

Cells were mounted with ProLong Gold Dapi mounting medium (Invitrogen) and visualized using a Zeiss LSM 510 confocal laser scanning microscope equipped with an Axiovert 100 M (Carl Zeiss, Jena, Germany).
